# Hierarchical Network Architecture for Non-Safety Applications in Urban Vehicular Ad-Hoc Networks

**DOI:** 10.3390/s19194306

**Published:** 2019-10-04

**Authors:** Sangsoo Jeong, Youngmi Baek, Sang Hyuk Son

**Affiliations:** Department of Information and Communication Engineering, DGIST, Daegu 42988, Korea; 88jeongss@dgist.ac.kr (S.J.); son@dgist.ac.kr (S.H.S.)

**Keywords:** hierarchical architecture, VANETs, cellular networks, Wi-Fi Direct, heterogeneous networks, non-safety information

## Abstract

In the vehicular ad-hoc networks (VANETs), wireless access in vehicular environments (WAVE) as the core networking technology is suitable for supporting safety-critical applications, but it is difficult to guarantee its performance when transmitting non-safety data, especially high volumes of data, in a multi-hop manner. Therefore, to provide non-safety applications effectively and reliably for users, we propose a hybrid V2V communication system (HVCS) using hierarchical networking architecture: a centralized control model for the establishment of a fast connection and a local data propagation model for efficient and reliable transmissions. The centralized control model had the functionality of node discovery, local ad-hoc group (LAG) formation, a LAG owner (LAGO) determination, and LAG management. The local data propagation indicates that data are transmitted only within the LAG under the management of the LAGO. To support the end-to-end multi-hop transmission over V2V communication, vehicles outside the LAG employ the store and forward model. We designed three phases consisting of concise device discovery (CDD), concise provisioning (CP), and data transmission, so that the HVCS is highly efficient and robust on the hierarchical networking architecture. Under the centralized control, the phase of the CDD operates to improve connection establishment time, and the CP is to simplify operations required for security establishment. Our HVCS is implemented as a two-tier system using a traffic controller for centralized control using cellular networks and a smartphone for local data propagation over Wi-Fi Direct. The HVCS’ performance was evaluated using Veins, and compared with WAVE in terms of throughput, connectivity, and quality of service (QoS). The effectiveness of the centralized control was demonstrated in comparative experiments with Wi-Fi Direct. The connection establishment time measured was only 0.95 s for the HVCS. In the case of video streaming services through the HVCS, about 98% of the events could be played over 16 frames per second. The throughput for the streaming data was between 74% to 81% when the vehicle density was over 50%. We demonstrated that the proposed system has high throughput and satisfies the QoS of streaming services even though the end-to-end delay is a bit longer when compared to that of WAVE.

## 1. Introduction

A cooperative intelligent transportation system (C-ITS) is a complex transport system with the cooperation between vehicles and specialized equipment located along transport infrastructure. It aims to provide ITS services for road users including drivers, passengers, and pedestrians so that they can experience better road safety, comfort, and improved mobility. The development of new technologies in wireless communication begins to motivate and drive change within C-ITS. It also leads to a growing interest in developing a wide range of applications based on vehicle-to-vehicle (V2V) and vehicle-to-infrastructure (V2I) communication. As one of the core networking technologies of C-ITS, there is wireless access in vehicular environments (WAVE). Through WAVE between on-board units in vehicles and road-side-units located along the transport infrastructure, drivers are informed in a timely fashion about the upcoming traffic situation so that they can take the necessary actions to avoid potential accidents. In this way, WAVE contributes to reduced congestion and improved driver comfort [1]. Moreover, the development of new technology allows drivers to extend an automobile from the main means of transportation as a new life space. First, these days, users in the vehicle are highly interested in enjoying entertainment (e.g., watching video streaming and playing games with friends) and utilizing various information for convenience and comfort. In this regard, during traveling, infotainment data have already been provided through network to vehicle (N2V) communication, for example, Internet access and a cellular network to vehicles [2,3,4]. Second, they are also interested in a traffic service providing real-time traffic information of the road in order to identify the cause when the road is congested. In this regard, in Korea, drivers can already receive real-time video streaming from CCTV installed along highways over a wired network or a cellular network (i.e., N2V or I2V) [5]. 

These non-safety services rely entirely on transport and wireless communication infrastructures, and I2V communication. In other words, the availability of this massive data transfer service may be limited to the area of the installed equipment. Its usability may also be limited by the communication costs that may vary depending on whether it is a pay-as-you-go model or a fixed payment model. It is expected that a large-scale deployment is possible soon—by 2021—to deploy 95% of WAVE-enabled devices on the real road [6]. V2V communication will be prevalent on the road in the near future. This may cause new demands for such non-safety services related to multimedia, regardless of the limitations of the fixed CCTV and additional cost. Consider an example of multimedia information services over WAVE-based V2V communication. In urban areas, many vehicles are at a near standstill in a traffic jam where some drivers are interested in watching the same video. If one of them receives multimedia data from the server first through the Internet, it can be a source for relaying the information over V2V communication [7,8]. Suppose that the vehicles’ black box should always record driving situations while traveling and is capable of uploading the recorded files continuously to a cloud server. Similarly, like the example of the multimedia information services and Korean traffic service above-mentioned, the video of the real-time traffic situation can be transmitted to the remote vehicle interested in situations where an accident or congestion has occurred in a specific area. If one of the vehicles interested in the same information is first provided with it from the infrastructure, it can be a source for relaying the information through WAVE-based V2V communication.

WAVE, which is specified in the IEEE 1609 family of standards, operates on the DSRC (dedicated short range communication) spectrum of 5.9 GHz. It is based on the IEEE 802.11p that defines functions for media access control (MAC) and physical layers. It is designed to support not only safety-critical applications by transmitting safety messages quickly and reliably on control channels, but also non-safety applications by transmitting either general user messages or IP traffic on service channels. It is expected that its performance will degrade as the number of vehicles demanding non-safety information such as video streaming and videoconferencing increases. In particular, real-time applications typically require stringent bandwidth and delay guarantees to satisfy an acceptable level of quality of service (QoS). However, it lacks the mechanisms to tackle performance degradation. In WAVE, the contention for wireless channel access makes it difficult to guarantee the quality of the streaming services and the reliable transmission of safety messages when the number of vehicle nodes and flows related to the real-time applications increase. Hence, there have been several attempts to support V2V communication without using WAVE. Some have tried to integrate various wireless networking technologies without depending on expensive transport and wireless network infrastructures [9]. For instance, because of the high penetration rate, smart mobile devices are considered as a viable option to implement V2X solutions [10,11]. Smart mobile devices such as smartphones, tablets, and smartwatches support a variety of wireless networking technologies including cellular communication, Bluetooth, NFC, Wi-Fi, and Wi-Fi Direct. There is great potential in these technologies to be capable of providing V2V communication as a substitute for WAVE in non-safety applications [12]. Without additional communication and purchase costs, they will be effective for providing more convenient and safer driving conditions on the road. Among these wireless technologies, there is no doubt that a cellular technology (including 3G, 4G, and 5G) is a good candidate for V2X communication because it provides wide coverage and reliable connectivity with low latency and a high data rate. The cellular technology is based on a centralized communication approach where a base station provides transmission coverage [13]. Another candidate among the wireless technologies of mobile devices to be considered in C-ITS is Wi-Fi Direct. This allows Wi-Fi-enabled devices to make ad-hoc networks without the communication infrastructure. Exploiting this Wi-Fi Direct capability, there was a study on the application of a smartphone to V2V communication to broadcast alerting information [14]. In addition, there was a study that transmitted a large volume of data such as video surveillance through Wi-Fi Direct by tackling the limitations of the Wi-Fi Direct [15]. One of the benefits of using Wi-Fi Direct is that the end-to-end (E2E) delay is shorter than that of 4G [16]. It is possible to distribute the workload effectively across local networks and existing infrastructure networks if it is adopted in V2X communication for non-safety applications. 

In this paper, we investigated an effective and reliable method of transmitting large volumes of data related to non-safety applications with timeliness requirements. In this regard, the problems we proposed to solve can be formulated as follows:

- PROBLEM 1. *What are other wireless technologies suitable for use instead of WAVE when transmitting non-safety data using a smartphone in urban vehicular ad-hoc networks (VANETs)?*
- PROBLEM 2. *Is a distribution approach sufficient for inter-vehicle communication transferring large volumes of non-safety data in time?*
- PROBLEM 3. *How should a group be formed quickly for the local transmission of the non-safety data?*
- PROBLEM 4. *How can distributed groups, which are highly affected by road structures such as intersections, byways, and traffic lights and the high mobility of vehicles, be maintained as long as possible in urban VANETs?*
- PROBLEM 5. *How can a relay node be chosen optimally for local transmission within a formed group with a specific topology?*
- PROBLEM 6. *Can smartphone-based wireless communication technology be an alternative to WAVE-based V2V communication?*

To answer these questions, we proposed a novel hybrid V2V communication system (HVCS) for non-safety applications in urban VANETs with the aim to transmit video data efficiently while satisfying the QoS with high connection reliability. To achieve this, we designed a hierarchical network architecture with a two-tier system that consisted of a centralized control model and a local data propagation model. Our hierarchical network architecture is capable of efficiently supporting many vehicular nodes by forming an optimal group with locality, establishing a rapid connection, and transmitting large volumes of data. The proposed HVCS based on this architecture consisted of traffic controllers and vehicle nodes. In order to perform centralized control, the traffic controller collects and monitors the vehicular information required, and manages the connections among the vehicle nodes. After the vehicles form a group with the help of a traffic controller, they can communicate locally within a group to transmit large amounts of non-safety data. However, the dynamic nature of vehicles in VANETs and the structure of urban road networks make it difficult to guarantee end-to-end connectivity. To increase the reliability of the end-to-end transmission of multimedia streaming, multi-hop delivery is performed between vehicles using a store and forward model when a vehicle cannot immediately connect to the next hop. The HVCS utilizes the cellular network to monitor the movement information and the status information of the vehicle nodes. It employs Wi-Fi Direct for both intra-group transmission and the store and forward mechanism. To demonstrate the effectiveness of the proposed HVCS, we evaluated the performance of the HVCS with a large road network using the Veins simulator in terms of throughput, connectivity, and the level of the QoS. The results show that the HVCS with the hierarchical network architecture is a standalone and practical system for non-safety applications.

The contribution of our research is as follows. First, we propose a novel hybrid V2V communication system that can transmit large amounts of non-safety data. Considering the dynamic nature of VANETs, we designed a two-tier architecture with the centralized control model for fast connections and the local propagation model for end-to-end delivery over a multi-hop path. Second, since the proposed system operates simultaneously and independently with WAVE, it does not affect the performance of WAVE for safety-critical applications. In addition, it can remove the need for the transmission of non-safety data through WAVE. Therefore, WAVE may not experience the performance degradation caused by many data flows of which the size is large. Third, to the best of our knowledge, this is the first attempt to apply Wi-Fi Direct to dynamic vehicular environments without any structural modification. Note that Wi-Fi Direct is generally designed for stationary environments. Fourth, we tackle the challenges of providing high reliability and connectivity in the end-to-end delivery and eliminate the unnecessary channel contention for data transmission. Our local propagation model is effective enough to satisfy the QoS of multimedia streaming. It is known that the minimum criterion that allows consecutive scenes to be recognized in video is 16 frames per second (FPS) [17]. In this regard, the proposed system demonstrates that about 98% of the total streaming events meet this minimum criterion. Finally, the proposed HVCS is a cost-effective and practical system because we only used smart mobile devices and a widespread wireless networking technology without the expensive transport infrastructure.

The remainder of this paper is organized as follows. In Section 2, we describe the background including the advantages and disadvantages of wireless networking technologies and introduce existing studies of hybrid networks. Section 3 provides the requirements for designing our HVCS with the detailed description of HVCS using the hierarchical network architecture, and the prototype implementation is introduced in Section 4. In Section 5, we evaluate the performance of the proposed system and compare it with WAVE. Finally, Section 6 concludes the paper.

## 2. Related Work 

One of the global standards for C-ITS, that supports safety-critical and non-safety applications is WAVE [18]. It is designed to broadcast particular messages, for example, basic safety messages (BSMs), based on collision-sense multiple access with collision avoidance (CSMA/CA). In WAVE, the number of dedicated channels is limited to seven and BSMs are mainly transmitted on a control channel (CCH). Both network association and authentication processes are eliminated to transmit them quickly. In the case of non-safety applications such as real-time video relay, remote vehicle personalization and diagnosis, and parking availability, a service channel (SCH) is used to transmit the related data. Before sending non-safety data on the SCH, a group (i.e., WAVE basic service set) first needs to be formed on the CCH. This means that BSMs and non-safety messages are transmitted respectively on each channel while a node hops between at least two channels. In WAVE, it is difficult to guarantee road safety as the traffic density of the roads, the amount of data to be sent, or the number of flows increase [18]. In order to enhance the performance of WAVE in this environment, many studies have tried to combine heterogeneous wireless network technologies (WNTs) to construct a hybrid network.

### 2.1. Hybrid Networks for Vehicular Ad-hoc Networks

For safety-critical applications, the existing hybrid networks mainly aim to provide an effective method to form and manage groups (i.e. clusters), in order to solve the broadcast storm problem and to deliver BSMs quickly. In particular, several recent studies have proposed the combination of WAVE and the cellular network [19,20,21]. Their basic idea is to form a cluster and to exchange BSMs among members of the cluster through WAVE. These studies, however, differ in the transmission of messages between clusters. 

One approach is to determine a cluster header among members of the cluster, according to the received cellular signal strength that represents the ability to relay messages [19]. The cluster header is responsible for inter-cluster communication using cellular networks. As the control overhead required for the determination of a route is very high on WAVE, the occupancy rate of the BSMs on the channel is relatively low. The second approach takes a different method to transfer data through the cluster boundary node existing between two clusters in a multi-hop manner, if there is a cluster adjacent to another cluster [20]. It uses WAVE to communicate between clusters and to discover relay nodes, and to manage the overhead since the relay nodes consume the WAVE resources. If it cannot find any relay node nearby, it uses the cellular networks for inter-cluster communication. The third approach is to use a cellular network for inter-cluster communication [21]. In the third method, as the number of vehicles on the road increases, the amount of data across the cellular network also increases.

There have been studies using WAVE to transmit non-safety data and use the cellular network for the control required for data transmission [22,23]. One of them suggests using the cellular network to determine a packet delivery path to collect information required for the location service [22]. It eliminates a broadcast storm that may occur while looking for multi-paths at a network layer. Cellular base stations (BSs) determine a route much faster than WAVE because it can rapidly and constantly identify the location of the vehicle through cellular networks [22,24]. Shukla et al. focused on the determination of routes for data dissemination to provide drivers with Internet access [23]. To achieve network load balancing, cellular BSs investigate the bandwidth of all available paths and then determine a route. In these studies, since non-safety data are transmitted on WAVE, underlying problems remain such as high channel contention and severe packet collisions as the vehicle density and the number of flows increase.

There has been a study to provide infrastructure-based vehicle communication without using WAVE, for non-safety applications [25,26]. This study used WiMAX to download video data and share them with nearby vehicles through Wi-Fi [25]. Its effectiveness in VANETs needs further study as the performance was evaluated with a very simple experimental scenario. Another attempt also utilized infrastructure-based vehicle communication to provide seamless connectivity for vehicles using both access points (APs) of WiMAX and Wi-Fi on the urban road [26]. The performance of this approach is dependent on the number of APs installed along the urban roads and the time taken for handover. Wi-Fi has a short communication range of 50 m and the vehicles move at various speeds. Accordingly, handover occurs continuously. Since the handover that happens along the road should be performed in time before departing from the current AP coverage, the APs need to be installed densely along the road without interference. If not, in the worst case scenario, the connection may be broken for a while. Since the establishment of a fast connection is the most important requirement for data transmission in dynamic VANETs, WAVE is designed with the elimination of the association and disassociation process considering the mobility of the vehicle. However, they still perform these processes in a dynamic environment.

### 2.2. Candidates for Hybrid Wireless Networks

In this subsection, we introduce some of the wireless networking technologies available to smart mobile devices, especially smartphones, in order to find out particular technologies suitable for the transmission of large amounts of non-safety data such as real-time video streaming data in VANETs. Modern smartphones are capable of supporting near field communication (NFC), Bluetooth, cellular communication, Wi-Fi, and Wi-Fi Direct.

The topology of VANETs dynamically changes due to vehicle movements at various speeds toward different destinations and the urban road network including traffic signals and intersections. Guaranteeing connectivity is challenging in such an environment. The WNTs with short transmission range have difficulties in local communication with the dynamic topology. The short communication range of the NFC (<10 cm) is a fatal flaw for implementing V2V communication in VANETs.

Bluetooth low energy (BLE) among Bluetooth versions is one of the most widely used WNTs for local communication. To achieve low energy consumption, it operates in sleep mode most of time, but can transmit data up to 150 m in open fields by increasing the power consumption [27]. The assessment of the feasibility of BLE in VANETS was performed in [28]. Their results showed that the communication range of BLE is about 60~100 m while vehicles are moving. For a vehicle at a speed of 120 km/h, its communication range was too short to send data. In addition, the round trip time (RTT) of BLE was measured as approximately 100 ms in the environment where the receiving vehicle was 40 m apart from the sending vehicle. It can be used for non-safety applications (e.g., exchanging alarm messages and simple chat messages), but only supports a low data rate of one Mbps [29]. Therefore, it is difficult to transmit a large amount of non-safety data such as video streaming. The time necessary for the pairing process between new devices is also one of the main reasons why it may not be a reasonable candidate for VANETs. 

The cellular infrastructures for 3G and 4G have already been built and 5G is now at the forefront. The most important advantage of a cellular network is reliability. The latency variance and the packet loss rate of cellular networks are lower than other WNTs. Park et al. proposed a V2X development framework for cellular phones on Android OS [10]. If this system uses 5G, they will be able to support the safety application, at least in theory. However, in the presence of large amounts of data to be transmitted, even though non-safety services are available over a cellular network, using the cellular network is another matter for a user. The transmission cost of users may grow ever larger as the sent data volumes increase if they are not members of an unlimited mobile data plan with a high cost. Their ISPs (Internet service providers) are required to handle the expansion of the transmission infrastructure facilities with the explosion of these data. Nonetheless, the cellular network is a very good solution for V2V communication because it still has wide coverage and provides stable connectivity.

In the Wi-Fi series of smartphones, there are Wi-Fi Direct operating in an ad-hoc mode and wireless LAN in an infrastructure mode. In Wi-Fi Direct, since all data are transmitted without infrastructure, data never flow into the backbone network [14]. Therefore, Wi-Fi Direct is the most reasonable choice for V2V communication. Wi-Fi Direct is also suitable for heavy traffic transmission such as video streaming because its coverage and transmission rate is wider than other WNTs [15]. Table 1 shows that it is about ten times faster than WAVE. However, Wi-Fi Direct is designed for a stationary environment like other WLAN. Consequently, it takes a long time to establish a connection in ad-hoc networks and its transmission is performed only within the Wi-Fi Direct networks. It is not designed to support the multi-hop transmission over the configured networks.

## 3. A Hybrid V2V Communication System

In this section, we introduce a hybrid V2V communication system (HVCS) in detail based on our approach. The HVCS aims to reliably, efficiently, and rapidly transmit large amounts of non-safety data for multi-hop transmission, supporting cost-effectiveness (i.e., V2V communication) in urban VANETs. To clarify the non-safety applications with large volumes of the data, we used a particular target as one of the traffic information services, as mentioned in Section 1. There were some nodes that required downloading the same multimedia file such as a video file of the live CCTV or the black box monitoring a certain road section, which is displayed on a screen of the center console of the vehicle in real time during traveling. If we were to provide video streaming of 720p, which is the abbreviation of 1280 × 720 resolution and progressive scanning, for vehicles, one flow requires at least four Mbps of bandwidth for end-to-end transmission. 

In this paper, reliable transmission indicates delivering the data successfully from end-to-end in time. In the urban VANET, its topology changes dynamically due to time and space, depending on vehicle factors such as various vehicle density and various speeds and road network factors such as traffic lights, intersections, and crosswalks. This makes it difficult to guarantee the performance of V2V communication [30]. Therefore, there is a need for a method to provide reliable communication by stabilizing the topology change of the VANETs. We focused on two characteristics. First, the group of urban vehicles, driving in the same road segment, which was a section of the road separated by each intersection or traffic light, had the characteristic that they had similar movements in the same direction along the road. This helps vehicles form a group with one vehicle as its center and maintain it as long as possible. Second, considering road segments with high vehicle density or road segments near signalized intersections, moving vehicles in their own direction were more likely to meet the vehicles moving in the opposite direction. All vehicles in a given transmission range regardless of their moving direction can contribute to the reliable propagation of data to be sent to a destination node in any direction. We, therefore, tried to exploit these characteristics to increase opportunities for successful E2E delivery and increase the duration of connection within a group. 

The efficient transmission method should transmit massive data by minimizing redundancy in data transmission. In addition, it should not hinder the delivery performance of the data for the safety-critical applications through WAVE. First, WAVE is a WNT that primarily not only supports safety-critical applications, but also allows vehicles to exchange the data of non-safety applications [31]. With WAVE, the packet delivery ratio under high vehicle density in urban roads is dramatically reduced [18]. High vehicle density occurs during commute time as well as near convention centers and shopping centers where the vehicle density becomes high locally and temporally. The high density causes a lot of contention and collisions, long transmission delay, and bandwidth consumption, which may affect the delivery of the safety messages (i.e., BSMs) through WAVE that need to be transmitted in time. Furthermore, if the non-safety data with a relatively larger volume than the safety data coexist with BSMs over WAVE, they add to the problems above-mentioned. Therefore, it is reasonable to use a WNT other than WAVE to support massive data delivery services among non-safety applications. Second, while the broadcast is intended to enhance the reliability of the transmission of BSMs, duplicate data overflow into the network. It is very inefficient in non-safety applications with large volumes of data. To take advantage of the broadcast, data should propagate in a local broadcasting manner to increase the level of concurrency of the data transfer. Hence, we employed peer-to-peer communication within intra-group and inter-group communication, considering that as the number of group members increases (i.e., the higher density of the group), the communication quality decreases.

The rapid transmission method provides seamless connectivity among adjacent vehicles. The most important requirement in multimedia data transmission is that these data should be delivered in a timely manner since they are meaningless over time. For fast delivery services of large amounts of data through V2V communication in urban VANETs, it is necessary to simplify the connection process as in WAVE to minimize the number of control packets consumed in connection establishment, and to increase the occupancy rate of the transmitted data on the channel. Especially, among the non-safety applications supported by VANETs, certain applications require lots of data, for example, infotainment services. For a streaming service to be realized effectively through V2V communication, multi-hop transmissions between end nodes should be supported, meeting the timeliness requirement. 

To achieve the cost-effectiveness, it is desirable to exploit the existing wireless communication infrastructure during data transmission. In addition, rather than requiring users to purchase a new device for vehicular communication, leveraging existing devices is much more effective. Note that smartphones are the most widely used among smart mobile devices that support wireless connections [32].

### 3.1. Systems Overview

We propose an HVCS with a hierarchical network architecture to improve the performance of video data transmission in VANETs. The hierarchical network architecture represents a two-tier system where there is a centralized control model in the upper layer and a local data propagation model in the lower layer. The HVCS performs centralized control for the establishment of a fast connection between vehicles before data transmission. To provide large amounts of data reliably and efficiently, a local data propagation model, which is the basis of multi-hop transmission, was applied. The local data propagation is referred to as transmissions within a local ad-hoc group (LAG). We utilized both cellular networks for the centralized connection control and Wi-Fi Direct for the local data propagation. 

**Figure 1:**
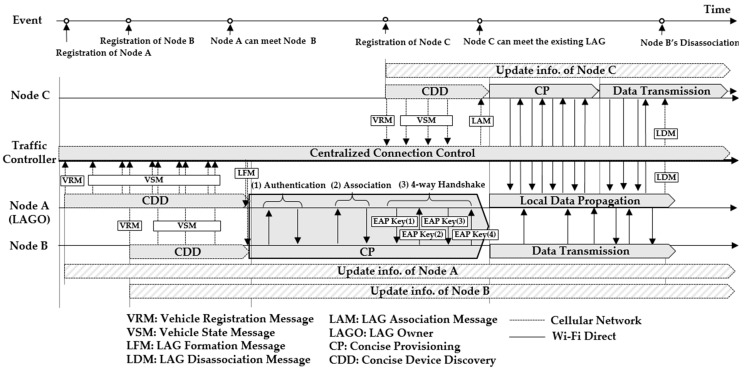
Overview of the proposed hybrid V2V communication system.

Figure 1 presents an overview of the proposed HVCS with the hierarchical network architecture. The three phases of HVCS, in which both a traffic controller and a vehicle node participate, consist of concise device discovery (CDD), concise provisioning (CP), and data transmission. The CDD is a phase of searching for an adjacent node in order to perform the connection control. The CP is a phase of establishing security between members within a given LAG for the data transmission. In the CDD, the traffic controller and nodes continuously participate using the cellular network as shown in Figure 1. In the CP and data transmission, only the nodes participate over Wi-Fi Direct. The traffic controller is responsible for centralized connection control, which performs tasks such as discovering nodes, determining the group (i.e., LAG), and informing the LAG of its decision. Therefore, the traffic controller does not intervene in the CP and the local data propagation of nodes. The CDD of node A starts at the time when node A is driving on the road. At the beginning of its CDD, node A sends its VRM (vehicle register message) to register with the traffic controller. After sending the VRM, node A continuously transmits VSMs (vehicle state messages) to the traffic controller in order to update their information during driving. These messages are described in detail in Section 3.3.2. When node B drives, its CDD starts with the same procedures as node A. If the traffic controller finds out that two nodes are approaching, the CP of nodes A and B starts. The traffic controller transmits LFMs (LAG formation message) with the members (i.e., node B) and node A as a local ad-hoc group owner (LAGO) of the LAG to be formed. Using the information on LFMs received over the cellular network, nodes A and B are grouped in the CP over Wi-Fi Direct. After the CDD and CP, the LAG is formed consisting of a LAGO and members. The LAGO is responsible for local data propagation within the LAG. During the CP of nodes A and B, the CDD of node C starts with the same procedures as nodes A and B. Once the traffic controller finds that node C approaching the formed LAG after a certain time, it transmits LFMs to the node C and the LAGO of the LAG. Node C becomes a member of the LAG through the CP. Under the centralized control, any member who leaves the given LAG may be quickly associated with another LAG and transfer its data between LAGs. At that time, the traffic controller uses LDMs (LAG dissociation messages) to inform the node’s withdrawal.

We assumed that the smartphones in the vehicles were directly connected to vehicles through connected solutions and wired connection. It was also assumed that the operation of the HVCS was continuous since battery-powered smartphones are continuously charged in the vehicle. Since the node consumes the maximum power in transmission, performance degradation caused by noise should be negligible. Each LAG can be formed, not only with any node moving in the opposite direction to disseminate data in as many directions as possible, but also with any node in the same direction to continue the connectivity as long as possible.

### 3.2. Cellular-Based Centralized Control Model

Due to the dynamic environment of the VANET, it is difficult to maintain a stable state for a locally formed ad-hoc network. When a LAG is formed under such an environment, it is possible that there are many trials to form a new LAG and/or association and disassociation of members within the LAG is highly frequent. It may take more time to establish a connection than the time for data transmission. This is why we used centralized control in our HVCS to manage the nodes for small LAGs and form a small LAG. The term *small* indicates that a LAG is designed to have a limited size. This is because there is a high potential for channel collisions as the node density within a given area becomes high if too many nodes contribute to forming a LAG. 

Note that a LAGO of our HVCS differed from a cluster header in sensor networks in terms of roles. First, a cluster header is generally designed to overcome performance degradation derived from energy constraints of sensor nodes [33,34]. A cluster header plays an important role in performing inter-group and intra-group communication, routing, and the member management of a given cluster. In contrast with a cluster header, a LAGO is not responsible for management because a traffic controller contributes to forming a local group, continues to manage the members of a given LAG, and helps the nodes establish new connections easily even when the designated LAGO has changed. Therefore, the designated LAGO focuses on transferring data between LAGs as well as relaying data within its own LAG. Second, to prolong the sensor network’s lifetime and maintain connectivity in a dynamic environment, determining an optimal cluster header in a given cluster is critical. A cluster header is mainly selected based on the fact that the transmission power of a node is directly related to wireless link quality and transmission range [35]. However, functions concentrated on a cluster header not only incur a high overhead, but also consume a large amount of energy of the cluster header even though an optimal cluster header is selected. In contrast, in our HVCS, the transmission power is no longer an important factor for the determination of a LAGO. It can provide richer connectivity because all nodes are being continuously charged in the vehicle. This means that it can achieve an acceptable performance for transmission even if a traffic controller determines anyone to be a LAGO among the members of the small LAG. The most important factor in V2V communication is fast LAG formation and rapid recovery from LAG splitting or corruption on the road. Furthermore, determining an optimal cluster header is known to be a NP-hard problem [33]. For this reason, we adopted a quick and simple heuristic method that used both random selection and distance-based determination when the traffic controller randomly determines a LAGO among LAG members.

The traffic controller is responsible for the CDD in which the vehicle node information is collected through the cellular network for searching the nodes and connection control. A traffic controller continuously maintains and monitors the vehicle node information that includes the device’s MAC address, available Wi-Fi channel data, and GPS information. Using this information, the traffic controller determines a LAGO node for the LAG formation, based on three basic rules as follows:

(1) When there is only one node, it cannot form a LAGO and should wait for new nodes.
(2) Given that δ denotes the maximum number of the members for the LAG, a new node can join the existing LAG only if it does not exceed δ, the maximum size of the LAG.
(3) A traffic controller performs distance-based control when constructing LAGs and determining their LAGOs with adjacent nodes, except that there are only two adjacent nodes. If there are only two nodes for a new LAG, one of the nodes is randomly determined as a LAGO, namely, random LAGO selection.

The distance-based control was used to construct a LAG, determine a LAGO among the nodes of the LAG, and form a new LAG again whenever the existing LAG is corrupted. The distance-based control was designed to provide as much connectivity as possible without isolated nodes on the road. This was also based on the fact that the communication quality responds to the transmission range of nodes. 

For the distance-based control, the basic idea is to find the median point of nodes for LAG formation, and then determine a particular node closest to that point as a LAGO. Following this process, a LAG is constructed with a star topology around the designated LAGO and is formed based on the transmission range of a LAGO. Therefore, the problem of LAG formation for the adjacent nodes is transformed into that of the LAGO determination. To define the problem of the LAGO determination, we considered N vehicles that drive in a vehicular network. Let V denote the set of all nodes $V=\left\{v_{1},v_{2,}\ldots ,\;v_{N}\right\},\;\;v_{i}\in {\mathbb{R}}^{2}$ (2-dimensional Euclidean space) that drive on the road. Vehicle $v_{i}\;$ is the i-th node, i∈N={1, 2, …, N}. Let $G_{l}$, which represents a LAG designed by a traffic controller, denote the set of all members including a LAGO node of LAG l,  l∈ℒ={1, 2,…,L}. The overall set of all the LAGs (i.e., the LAG space) is $G={\cup^{\;}}_{l\in ℒ}G_{l}$. Given a formed LAG $G=\left\{g_{1},g_{2,}\ldots ,\;g_{K}\right\}\in G,$ $g_{i}$ is the member belonging to LAG. Among them, let $g_{i}^{*}$ denote the LAGO i of the LAG and $g_{-i}^{*}$ denote the other members of LAG except for LAGO $g_{i}^{*}$, which is the i-th node, i∈K={1,2,…,K} and K⊂N. In this paper, the LAGO determination was formulated as follows:

𝒫 : < ℒ, {*S_l_*}, {*u_l_*} >, (1)

- ℒ is the set of LAG identifiers. LAG l is the l-th LAGs generated, l∈ℒ
- $S_{l}\left(l\in ℒ\right)$ is the set of the k candidates for LAG l. The traffic controller chooses the available nodes from set V. The overall node set of all LAGs (i.e., the candidate space of the LAGO determination) is S = U_*l*∈ℒ_*S_l_*.
- $u_{l}$: S→V(l∈ℒ) is the determination function of the LAGO of LAG l. At a certain time, for the LAG formation of the candidate list $\left\{s_{1},s_{2,}\ldots ,\;s_{k}\right\}\in \;S$ and k∈N, $u_{l}\left(s_{1},s_{2,}\ldots ,\;s_{k}\right)\in \;V$ is the node determined as a LAGO in the LAG l with $s_{i}\in S_{l}(i\in K$ and l∈ℒ).

Given a candidate set $S=\left\{s_{1},s_{2,}\ldots ,\;s_{k}\right\}\in S$, $s_{i}$ indicates the node $v_{i}\;\left(i\in K\;and\;v_{i}\in V\right)$ within a transmission range where local communication is possible on the road. Under the management of the traffic controller, the determination of LAGO in the LAG l is to choose a LAGO node with adjacent nodes. That is, $g_{i}^{*}≔v_{i},\;v_{i}\in V$. Node i is determined as a LAGO only when it has the minimum value of the determination function $u_{l}$. The determination function of LAG l keeps the Euclidean distances from each point ${\left\{s_{i}\right\}}_{1}^{k}\in {\mathbb{R}}^{2}$ and is given as:

(2) $$u_{l}\left(s_{1},s_{2,}\ldots ,\;s_{k}\right)\underset{s_{i}}{=\mathrm{argmin}}\|\mu -s_{i}\|{}_{2},$$

where $s_{i}$ represents a candidate node that is available to form a LAG (i∈K); ‖·‖ denotes the Euclidean norm, and μ ($\mu \in {\mathbb{R}}^{2})$ is the mean point among the k candidate sets ${\left\{s_{i}\right\}}_{1}^{k}\in S$ and $\in {\mathbb{R}}^{2}$. In the space of ${\mathbb{R}}^{2}$, the mean point μ  of the k candidates ${\left\{s_{i}\right\}}_{1}^{k}\in {\mathbb{R}}^{2}$ can be calculated by:

(3) $$\mu =\left(P_{\hat{x}},\;P_{\hat{y}}\right)=\left(\frac{\sum_{i=1}^{k}P_{x}\left(s_{i}\right)}{n},\frac{\sum_{i=1}^{k}P_{y}\left(s_{i}\right)}{n}\right),$$

where $P_{x}\left(s_{i}\right)$ is the coordinate for the horizontal *x*-axis of the node $s_{i}≔v_{i}$ and $P_{y}\left(s_{i}\right)$ is the coordinate for the vertical *y*-axis of the node. By using Equations (2) and (3), the LAGO is determined as the node with the shortest distance from the mean point.

#### 3.2.1. LAG Formation and LAGO Determination

Here, we considered several cases. First, a traffic controller monitors several nodes but cannot form a LAG if there is only one node $v_{i}\;$ on the road and there are no other nodes to connect to. Second, if there is no LAG on a certain road segment and one node $v_{j}$ is newly adjacent to existing node $v_{i}\;$ within a transmission range in which local communication is possible on the road, the traffic controller randomly selects one $v_{i\left(i\in N\right)}\;$ of them as a LAGO (i.e., random LAGO selection). Therefore, a new LAG is designated as $G_{l1}=\left\{g_{i},\;g_{j}\right\}$ with LAGO $g_{i}^{*}$. If there is no LAG and one or more nodes are newly present at the same time and are adjacent to each other, the LAGO is determined by using the LAGO determination of the distance-based control of Equations (1) and (2). In Algorithm 1, we present these processes to describe how to perform the LAG formation and LAGO determination by a traffic controller in detail.

Third, if there is one LAG on a certain road segment and one node $v_{k}$ is newly within transmission range of the LAGO of the existing LAG $G_{l}$, the LAG becomes $G_{l}=\{g_{i}^{*},g_{j},g_{k}\}$. Finally, if one or more nodes are newly adjoined to each other, but they are not adjacent to existing LAG $G_{l},$ depending on how many nodes are adjacent, the traffic controller can perform either random LAGO selection or LAGO determination of the distance-based control using Equations (2) and (3). Until the size of the LAG reaches at δ, the traffic controller manages a new node to join the existing LAG throughout the distance-based control. Therefore, if it is full of the members as δ when a new node is adjacent to the existing LAG, the new node cannot join and should wait for another node. We describe these processes, namely, the LAG joining process, in Algorithm 2.


**Algorithm 1: New LAG Formation and LAGO Determination**
Input: Known S, the set of the k candidates for the formation of LAG l (k≥1)
Output:The member nodes and a LAGO node in LAG l
1**IF**|S|==1 (i.e., k
== 1)2  Wait3
**Else If**
4  Calculate the mean point μ among $S=\left\{s_{1},s_{2,}\ldots ,\;s_{k}\right\}$
5  Determine LAGO $g_{i}^{*}\;$ by using $u_{l}\left(S\right)$ with the mean point μ
6  Take the LAG $G_{l}=\left\{g_{i}^{*}\;,\;g_{-i}^{*}\right\}$
7  Send LFM to LAGO $g_{i}^{*}$
8  **For** each vehicle $s_{i},$
**do**9    **If**
$s_{i}\neq$
$g_{i}^{*}$10      Send LAM($g_{i}^{*}$) to $s_{i}$
11    **End If**12  **End For**13
**End If**


After the LAGO determination, the traffic controller transmits a LAG formation message (LFM) to nodes of the LAG $G_{l}\;\left(l\in ℒ\right)$, which is a message instructing them to construct a new LAG. If a node is determined to join the existing LAG, the traffic controller forwards a LAG association message (LAM) to help new nodes join the LAG just as they approach the LAG that already exists.


**Algorithm 2: LAG Joining Process**
Input: Known $G={\cup^{\text{​}}}_{l\in ℒ}G_{l}$, $G_{l}=\left\{g_{l}^{*}\;,\;g_{-l}^{*}\right\}\;$ as the set of the nodes of the existing LAG l
(l∈ℒ) Known S, the set of the k candidate nodes Known δ, the maximum number of the members for the LAGOutput:The member nodes and a LAGO node of the selected LAG l
1Take a set of LAGOs $G_{}^{*}=${$g_{1}^{*},g_{2}^{*},\ldots ,g_{l}^{*}\}$ from all LAGs $G={\cup^{\text{​}}}_{l\in ℒ}G_{l}$
2Define a distance set D=∅
3
**If**
$|G_{}^{*}|==1\;$
4  If $\;\left|G_{l}\;\left(g_{i}^{*}\in G_{}^{*}\right)\right|$ < δ
5    Send LAM to LAGO $g_{i}^{*}$
6    Send LAM($g_{i}^{*}$) to $s_{i}$
7  **Else**8    Wait 9  **End If**10
**Else**
11  **For** each vehicle $s_{i}$
**do**12    **For** each LAGO $g_{j}^{*}$
**do**13      Calculate the distance $\rho \left(g_{j}^{*},s_{i}\right)$ between vehicle $s_{i}$ and {$g_{1}^{*},g_{2}^{*},\ldots ,g_{l}^{*}\}$ in all LAGOs; $\rho \left(g_{j}^{*},s_{i}\right)=\|g_{j}^{*}-s_{i}\|{}_{2}$
14      Take the distance set $D\cup^{\text{​}}\left\{\;\rho \left(g_{j}^{*},s_{i}\right)\right\}$
15    **End For**16    According to D, after getting the ascending order of D, take $g_{m}^{*}\;$ with the minimum distance from $s_{i}$
17    Send LAM to LAGO $g_{m}^{*}$
18    Send LAM($g_{m}^{*}$) to $s_{i}$
19    Define D=∅
20  **End For**21
**End If**


#### 3.2.2. LAG Disassociation and LAGO Corruption

First, if any node $g_{x}\left(x\in K\right)$ belonging to the LAG $G_{l}\;=\left\{g_{k},g_{i}^{*}\ldots ,\;g_{m}\right\}\;$ is out of the local transmission range of the LAGO $g_{i}^{*}$, the traffic controller delivers a LAG disassociation message (LDM) to all members of the LAG and that node $g_{x}$. This is available because the traffic controller continuously monitors the set of vehicles $V=\left\{v_{1},v_{2,}\ldots ,\;v_{N}\right\}\;\in {\mathbb{R}}^{2}$. By doing this, the LAGO can be aware of all members of the LAG in a consistent manner and prevent the data transfer from any member of the LAG to the disassociated member $g_{x}$. Second, the existing LAG $G_{l}\;$ collapses in the case where all members $g_{-i}^{*}$ move in the same direction, but only LAGO $g_{i}^{*}$ moves to the other direction. To recover connectivity among remaining nodes from this corruption rapidly, the traffic controller performs the LAG formation and the LAGO determination of Algorithm 1 with them, as discussed earlier. If there is only one member left after the LAGO $g_{i}^{*}$ leaves the LAG, it cannot make a connection to any node and should wait for another node according to Algorithm 1. However, if the node is adjacent to the existing LAG, in order to continuously provide connectivity for that node, the node can join the existing LAG by using distance-based control until its number reaches δ. This process, called LAG disassociation, is presented in Algorithm 3. 

Finally, if the traffic controller does not receive on three consecutive times the vehicle node information from a certain node $g_{x}\left(x\in K\right)\;$ belonging to the LAG $G_{l}\;=\left\{g_{i}^{*},\;\;g_{-i}^{*}\right\}$, according to a role (i.e., $g_{i}^{*},\;\;g_{-i}^{*})\;$ of the node within the LAG, the traffic controller performs either Algorithm 3 for $g_{-i}^{*}\;$ or Algorithm 4 for $g_{i}^{*}$. We describe Algorithm 4 of LAGO corruption where the members of the LAG should rapidly form a new LAG since the existing LAG $G_{l}\;$ collapses due to the LAGO that is considered not to respond to the traffic controller.


**Algorithm 3: LAG Disassociation**
Input:Known $G_{l}$, LAG l
$G_{l}=\left\{g_{-i}^{*},\;g_{i}^{*}\right\},\;i\in K$ Known $g_{x}\in g_{-i}^{*}$, a node $g_{x}\;$ is out of the local transmission range of the LAGO $g_{i}^{*}$ or a non-response node Known $S={\cup^{\text{​}}}_{l\in ℒ}S_{l}$, $S_{l}\left(l\in ℒ\right)$ is the set of the candidate nodes Output:The member nodes and a LAGO node in LAG l
1Send LDM($g_{x})$ to $g_{x}$, $g_{i}^{*}$, and $g_{-i}^{*}-\{g_{x}\}$
2Send LDM($g_{i}^{*})$ to $g_{x}$
3Take $G_{l}=G_{l}-\left\{g_{x}\right\}$
4
**IF**
$|G_{l}|==1$
5  Take $s_{i}≔g_{i}^{*}\;and\;G_{l}=\emptyset$
6  Perform Procedure ***Setup***($s_{i}$)7
**End If**
8Take $s_{i}≔g_{x}$ and perform Procedure ***Setup***($s_{i}$) 9Perform Algorithm 1 or Algorithm 2 for S
10Take $S={\cup^{\text{​}}}_{l\in ℒ}S_{l}$
−
$S_{x}\;\mathrm{and}\;ℒ-\left\{x\right\}$
Procedure ***Setup*** ($s_{i}$) for Algorithm 3Input:Known $S={\cup^{\text{​}}}_{l\in ℒ}S_{l}$, $S_{l}\left(l\in ℒ\right)$ is the set of the candidate nodesOutput:The set of the k candidate nodes, $S_{}$ (k≥1)
1**IF**$S_{l}$ adjacent to $s_{i}$2  Take $S_{l}\;\cup^{\text{​}}\left\{s_{i}\right\}$
3
**Else**
4  Define $S_{x\left(\left\{x\right\}\cup^{\text{​}}ℒ\right)}=\emptyset$ and take $S_{x}\cup^{\text{​}}\left\{s_{i}\right\}$
5  Take $S={\cup^{\text{​}}}_{l\in ℒ}S_{l}$
$\cup^{\text{​}}$
$S_{x}$
6
**End If**

**Algorithm 4: LAGO Corruption**
Input: Known $G_{l}$, LAG l
$G_{l}=\left\{g_{-i}^{*},\;g_{i}^{*}\right\},\;i\in K$ Known $g_{i}^{*}\in G_{l}$, a LAGO $g_{i}^{*}\;$ is a non-response node Known $S={\cup^{\text{​}}}_{l\in ℒ}S_{l}$, $S_{l}\left(l\in ℒ\right)$ is the set of the candidate nodesOutput:The member nodes and a LAGO node in LAG l
1Send LDM($g_{i}^{*})$ to $g_{i}^{*}$ and $g_{-i}^{*}$
2Define $S_{x\left(\left\{x\right\}\cup^{\text{​}}ℒ\right)}=\emptyset$ and take $S_{x}\cup^{\text{​}}\left\{g_{-i}^{*}\right\}$
3Take $S={\cup^{\text{​}}}_{l\in ℒ}S_{l}$
$\cup^{\text{​}}$
$S_{x}$
4Perform Algorithm 1 for $S_{x}$
5Take $S={\cup^{\text{​}}}_{l\in ℒ}S_{l}$
−
$S_{x}\;\mathrm{and}\;ℒ-\left\{x\right\}$


#### 3.2.3. Key Generation

The traffic controller generates a master key that is used for the nodes to form a new LAG and to join an existing LAG, and for the security establishment in the CP described in Section 3.3.2. It puts the master key to the LFM and LAM. In the CP, the generated master key is used to generate a session key for secure data transmission. Note that the master and session keys are the pairwise secret keys only for both the LAGO and one member. Therefore, the traffic controller generates master keys for the number of members of the LAG. To generate a master key, the traffic controller performs node authentication based on the registered information of nodes. The authentication performed by the controller employs the registration protocol described in IEEE 802.11i [36]. A master key denoted as $MK_{LAGO,\;k}$ is given by concatenating the following information on behalf of the LAGO and a node k.

(4) $$MK_{LAGO,\;k}=\psi \left(\left[MRK_{LAGO,\;k}∥MSK_{LAGO,\;k}∥PAD\right]\right),$$

where ∥ indicates an operator of concatenation; $MRK_{LAGO,\;k}$ is a master receive key; $MSK_{LAGO,\;k}\;$ is a master send key; and PAD is the zeros padding of 32 bytes. The function ψ takes the most significant bits of 256 from the given concatenated data. We used the authentication, authorization, and accounting (AAA) server, which is a network server used for access control and a standalone system installed outside the traffic controller [37]. Exploiting the AAA server, the traffic controller can acquire the authentication information $MRK_{LAGO,\;k},\;\;MSK_{LAGO,\;k}$ required for generating the master key.

Nodes receiving the instruction from the traffic controller form a new LAG. On the road, every node should register itself by periodically uploading its information to the traffic controller. This registration period is determined to be equal to the GPS signal reception cycle (i.e., one second). If the information of the nodes is not updated every cycle, the traffic controller checks the update timestamp of the node information and deletes the registration information after a predetermined time. 

**Figure 2:**
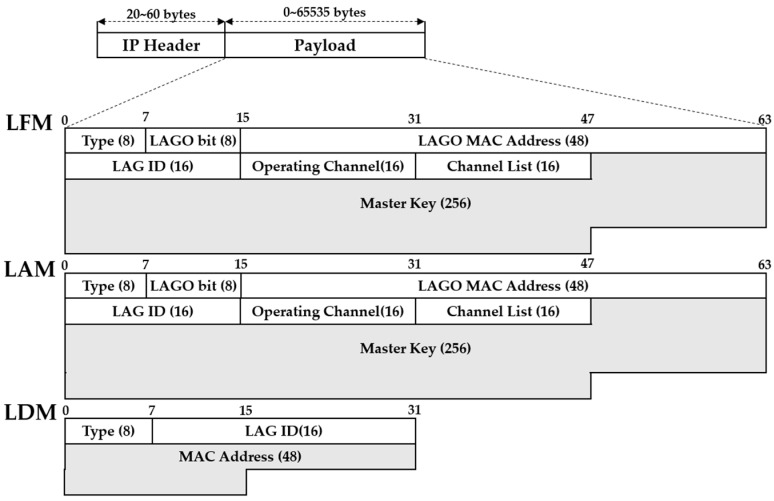
Messages of a traffic controller.

We defined three message formats as shown in Figure 2. The LFM is a message for forming a LAG and is defined using the following fields. The Type field indicates the message type and its value is one for LFM, two for LAM, and three for LDM. The LAGO MAC Address is the MAC address of the LAGO determined by a traffic controller and the value of the LAGO ID is given by taking the least significant bits of 16 from the LAGO MAC address. The value of the LAGO bit field is given as 15 for a LAGO. For a member of the LAG, the value of the LAGO bit is selected randomly in the range of 0 to 14. The Operating Channel field refers to the channels in which the LAG can be formed and its value is selected among the channels of 1, 6, and 11 to minimize interference with neighboring networks. Since the channels available for Wi-Fi Direct vary from country to country, the Channel List field informs the nodes of all available channels. If a node encounters the LAG, the LAM gives the information of the existing LAGO and LAG to the node. Therefore, the format of LAM is the same as that of the LFM. Since the LDM is a message for sending the MAC address of the leaving node to all members and the LAGO of the LAG, the LDM consists of only three fields: type, LAG ID, and a node’s MAC address.

### 3.3. Wi-Fi Direct-Based Local Data Propagation

The nodes receiving the LFM to form a new LAG or the LAM to join an existing LAG should perform the Wi-Fi Direct connection and security establishment steps. Once the LAG formation is complete, the associated nodes can exchange data through the LAGO of the LAG using Wi-Fi Direct. The fast LAG formation is required for the VANETs and is highly related to fast connection and security establishment. For this reason, we performed the CDD and CP as discussed earlier. The CP is a security setup to be used after the LAG formation by the LAGO and its members. It uses the master key of LFM or LAM provided by the traffic controller. The CDD and CP are designed to address the technical challenge of the connection and security establishment of Wi-Fi Direct. 

#### 3.3.1. Problems of Conventional Connection Establishment Procedure of Wi-Fi Direct

The results of the CDD and CP of our HVCS was the same as the result of the conventional connection establishment procedure of Wi-Fi Direct, but the process of obtaining the connectivity among nodes is obviously different. To clarify the distinction between them, we introduce the conventional connection process of Wi-Fi Direct and its problems in this subsection.

**Figure 3:**
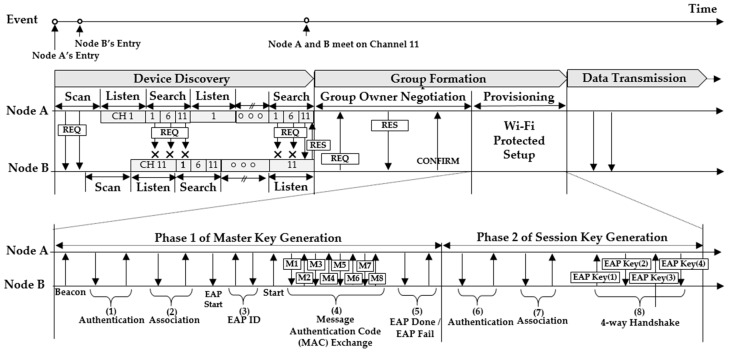
Wi-Fi Direct connection sequence.

The connection establishment phase of Wi-Fi Direct is shown in Figure 3 and is divided into device discovery (DD) and group formation (GF). The GF consists of group owner negotiation (GON) and provisioning to establish a secure connection and generate a session key.

The DD has three phases consisting of scanning, listening, and searching [38]. The objective of the scanning phase is to scan all channels and find the network in the vicinity. For the nodes to communicate, they start the DD themselves by sending the REQ (Request). In the listening phase, node A waits for a certain period in one channel (e.g., channel 1) and for packets (i.e., REQs) that any of the adjacent nodes sends in order to find it. On the other hand, in the searching phase, since node A searches for another node while traversing the specified channels (e.g., channels 1, 6, and 11), it may broadcast packets to other nodes of the listening phase. Node A repeats the listening and searching phases until another node. In other words, the repeat is over at a certain time when node A in the listening phase meets node B in the searching phase. In the GF, after they randomly determine a group owner, nodes A and B perform Wi-Fi protect setup (WPS) for security. The WPS not only generates a master key through mutual authentication based on the message authentication code, but also a session key in Phase 2 through a 4-way handshake. Finally, the generated session key is used as a key for intra-group communication. Figure 3 presents the process to generate the master key (Phase 1) and the process to generate the session key (Phase 2). 

In this paper, we defined the total time for DD and GF as connection establishment time (CET), which is the time required before exchanging data in a group. Camps-Mur et al. mentioned that from the experimental results, the CETs could take up to 15 s in the worst case [39]. In their study, the DD took 4 to 10 s and the WPS took 1 to 5 s, while the time spent for the GON was negligible in the CET. This indicates that its CET was too long to apply Wi-Fi Direct to dynamic environments to support the transmission of large data files. In addition, if any of the messages required is missed in each phase of the WPS, all processes are rolled back and should be restarted from the beginning of the phase. This also leads to a delay in connection establishment. Therefore, our HVCS eliminates the DD and the GON in the GF so that each node performs independently and allows the nodes to connect quickly under the control of a traffic controller. 

#### 3.3.2. Concise Device Discovery and Concise Provisioning

**Figure 4:**
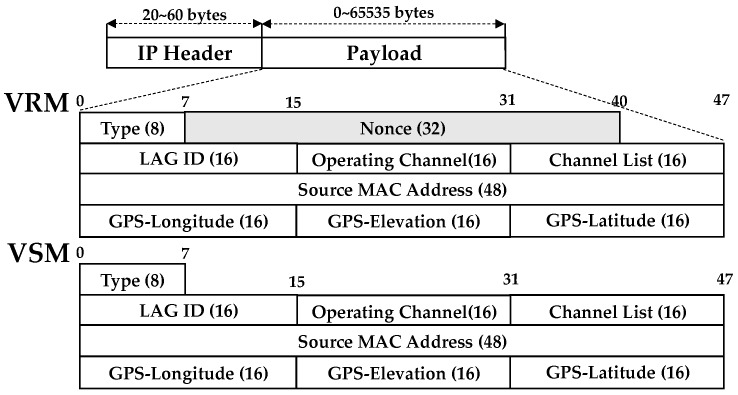
Messages of a vehicle node.

In order to participate in the CDD, the nodes should periodically transmit their information to the traffic controller as discussed in Section 3.2. For this process, a vehicle registration message (VRM) for initial registration and a vehicle state message (VSM) were defined as shown in Figure 4. The field of the source MAC address was the MAC address of the node to be registered, and the values of longitude, elevation, and latitude of the GPS were inserted into the corresponding fields.

After the nodes receive the LFM form a LAG without performing the GON of Wi-Fi Direct, the nodes enter the CP. The field of the master key of the LFM provides the pairwise key for the security establishment. Based on the received master key, the LAGO and the member node carry out authentication, association, and 4-way handshake to generate the session key. These processes follow those in Phase 2 of Wi-Fi Direct. The member node uses EAP-PSK to ask LAGO to authenticate it. After authentication, it is allowed to connect to LAGO. After performing the LAGO-driven 4-way handshake, the LAGO and the member k generate the same session key $\;SK_{LAGO,\;k}$ by concatenating the following shared information.

(5) $$SK_{LAGO,\;k}=\left[MK_{LAGO,\;k}∥\eta_{LAGO}∥\eta_{k}\;∥\Phi_{LAGO}∥\Phi_{k}\right],$$

where ∥ indicates an operator of concatenation; $MK_{LAGO,\;k}$ is the master key received from the traffic controller; $\eta_{LAGO}$ and $\eta_{k}$ are the nonce of the LAGO and the node k, respectively; and $\Phi_{LAGO}$ and $\Phi_{k}$ are the MAC addresses of the LAGO and the node k, respectively.

The session key is used to prove the integrity of the data as well as the credentials of the node when forwarding the data. It needs to be generated independently between the node and LAGO, and not the traffic controller. 

#### 3.3.3. Multi-Hop Data Transmission

In order to support multi-hop data transmission, we designed intra-LAG communication and applied a store and forward model (SAF) for inter-LAG communication. Our multi-hop data transmission aimed to enhance the reliability of data transmission and minimize redundant data transmissions. Therefore, it carries out local broadcast within LAG, and exploits the flow of the moving vehicles in the opposition direction. The LAGO has the responsibility for intra-LAG communication. The nodes other than the LAGO perform the SAF to achieve end-to-end data communication. Note that our method does not support inter-LAG communication based on LAGO. This is because Wi-Fi Direct operates in the ad-hoc mode but does not support transmission between the LAGOs. 

In intra-LAG communication, the source node sends a frame for video streaming to the LAGO. The LAGO delivers to the destination when the destination and the source are present at the same time in this LAG. If the destination exists in another LAG, the LAGO transmits the received frame to all LAG members in order to forward it to other nodes by using the SAF scheme. The node leaving the LAG performs inter-LAG communication. Once the member of the old LAG is newly associated with another LAG, it can deliver a certain amount of frames to the new LAGO. 

## 4. System Prototype Implementation

**Figure 5:**
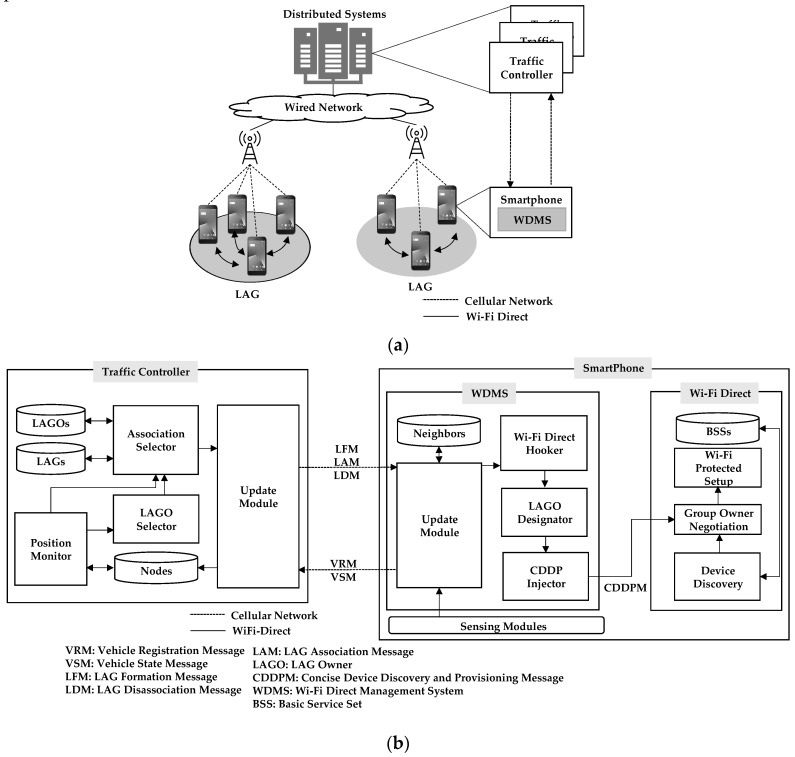
Design and implementation. (**a**) Overall structure of the implemented hybrid V2V communication system (HVCS). (**b**) Details of the traffic controller and the smartphone in the HVCS.

In this section, we describe how the proposed HVCS with a two-tier management was implemented on a traffic controller and smartphone. Figure 5 shows the overall structure of the HVCS and the details of the components of the two-tier system. As shown in Figure 5a, the smartphone of the vehicle makes local communication with members in the LAG through Wi-Fi Direct. On the road, smartphones are connected to our server as a traffic controller. For large scale implementation, a traffic controller should be a locally distributed system through cellular networks in order to reduce its load of the management of numerous vehicles. In the current implementation, there is only one traffic controller. The implementation structure of the traffic controller and the smartphone is presented in Figure 5b. The traffic controller consists of three databases (DBs) and four modules: the Nodes DB, LAGOs DB, LAGs DB, LAGO Selector, Association Selector, Location Monitor, and Update Module. 

The Update Module receives the VRMs and VSMs from the node, stores the related information in the Nodes DB, and transmits the LFMs, LAMs, and LDMs. To manage the vehicles, the Nodes DB consists of the vehicular information, which is periodically updated: a nonce, a MAC address, a location, a channel list, operating channels, and a LAGO ID. To manage the LAGOs, the LAGOs DB stores the LAGO’s MAC address, operating channel, and LAGO ID. The LAGs DB has LAG ID, the size of the LAG, and LAGO ID to manage LAGS. The size of the LAG is defined as the number of members including the LAGO. 

The LAGO Selector is a module that selects a LAGO when forming a LAG, and the Association Selector determines the LAG with which a given node will be associated. When a new node arrives within the transmission range of the LAG, the node is joined to that LAG. If the size of the LAG reaches the maximum number, it cooperates with the Location Monitor to create a new LAG. The Location Monitor is a module for identifying the encountered nodes within a Wi-Fi Direct transmission range and the nodes’ location and moving direction. We set the local transmission range to 200 m to form a LAG to use Wi-Fi Direct. Although it is the theoretical transmission range, some studies have demonstrated its performance in practice [40,41,42]. In our prototype, the maximum size of each LAG was limited to eight nodes, which is defined by the device manufacturers (e.g., Sony and BlackBerry) supporting Wi-Fi Direct, considering environmental interference [43,44]. 

We designed and implemented a Wi-Fi Direct management system (WDMS) of HVCS on a smartphone. The WDMS consists of Neighbors DB, CDDP injector, LAGO designator, Wi-Fi Direct hooker, and Update Module. The Update Module receives LFMs, LAMs, and LDMs from the traffic controller and stores LAG-related information in the Neighbors DB. The Neighbors DB has a list of members of the LAG to which this node belongs. 

**Figure 6:**
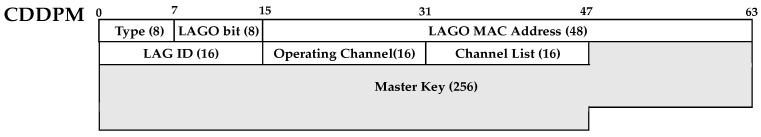
An internal message of a vehicle node.

Using the LFM, the LAGO designator determines whether this node is a LAGO or not, and generate a push message (i.e., a CDDP message (CDDPM)) to be sent internally to Wi-Fi Direct. The CDDP injector performs the function of pushing the CDDPM to the Wi-Fi Direct module. The information of the CDDPM is used to form the LAG and set up the security for data transmission over Wi-Fi Direct. The CDDPM is shown in Figure 6.

The Wi-Fi Direct hooker is a module that acts as a supervisor to take control of Wi-Fi Direct in operation and enables WDMS to be performed. After the CDDP injector completes pushing the CDDPM, this hooker hands over control to the Android OS so that the function of the Wi-Fi Direct is performed.

### 4.1. Feasibility Assessment of Forming LAG

We present our experiments using the actual implementation of the proposed HVCS. It demonstrates that, under the management of the traffic controller, the LAG is formed and the connections over Wi-Fi Direct are established among the smartphones in the vehicles traveling on the real roads. We conducted experiments on the real road with the developed prototype using three Microsoft Lumia 950xl smartphones and Windows systems in each of the three vehicles [38]. In these experiments, we used the LFM, LAM, and LDM of the proposed traffic controller. 

**Figure 7:**
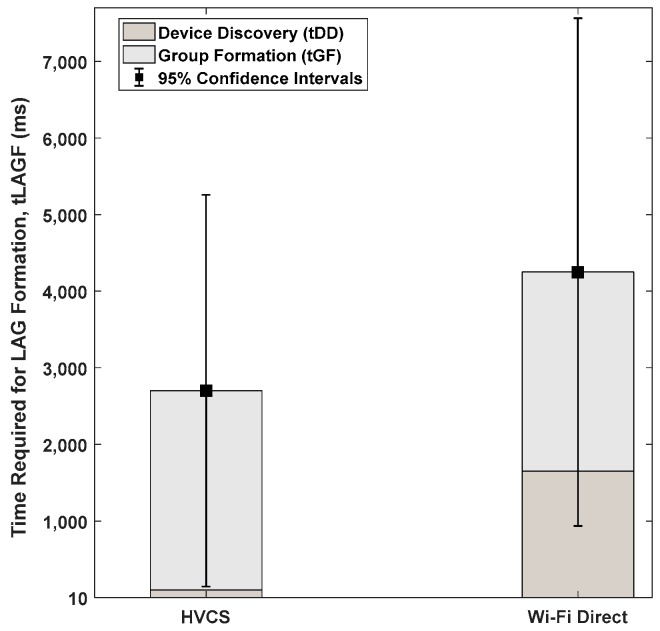
Comparison between the time taken for the LAG formation of HVCS and Wi-Fi Direct.

In this experiment, all of the three vehicles moved in the same direction. Two vehicles (i.e., $\nu_{1}$ and $\nu_{2}$) moved at the same speed of 20 km/h, but the other vehicle $\nu_{3}$ moved at the speed of 30 km/h. Since two vehicles $\nu_{1}$ and $\nu_{2}$ start driving very close to each other, two nodes form one LAG immediately and the LAGO is determined randomly between two vehicles. The other vehicle $\nu_{3}$ approaches this group of $\nu_{1}$ and $\nu_{2}$ with a faster speed than those of the two vehicles and then it joins the LAG. After a while, the LAGO goes out of range of the LAG with fast movement. The LAG formation time for each vehicle that drives on the actual road of DGIST in Korea was measured. We compared the LAG formation time of the nodes with that of Wi-Fi Direct. This experiment was performed in an environment where the traffic controller was not involved in determining a specific LAGO. The average LAG formation time of nodes denoted as tLAGF can be defined as follows.

(6) $$tLAGF=\;\frac{\sum_{i=1}^{n}\sum_{j=1}^{m}tDD_{i}^{j}+tGF_{i}^{j}}{n},$$

where n is the number of nodes, and m is the number of formation, which was two in this experiment. tDD is the amount of time taken for the node discovery, and tGF is the amount of time taken for the LAG formation and security establishment. In Figure 7, we can see that the tGF of our prototype was very similar to Wi-Fi Direct. However, our tDD was 15 times lower than that of Wi-Fi Direct due to the centralized control. The results show that by using a smartphone with a prototype of HVCS enabled, it is possible to connect with another vehicle’s smartphone while driving, and demonstrate that it actually operates on the real road.

## 5. Evaluation

In this section, for performance evaluation, we used the Veins simulator that integrates the traffic simulator Sumo with the networking simulator OMNet++ [45,46]. Our experiments using the Veins simulator were conducted with two scenarios. The first scenario aimed to show how long it takes to form connections among vehicles using the HVCS when n nodes establish connections at the same time, compared with that of the Wi-Fi Direct. This was conducted with a large scale of vehicles in a small map with 400 m × 400 m since only three of our HVCS prototypes were involved in the experiment described in Section 4.1. The experiment under the second scenario was performed to show the performance of the end-to-end transmission of the video streaming service in a large map with 1000 m × 2000 m. This experiment is concerned with demonstrating the effectiveness of the proposed HVCS by evaluating the performance of HVCS with a large road network in terms of the throughput, connectivity, timeliness, and QoS, and compare them with those of the WAVE. 

### 5.1. Connection Establishment Time 

**Figure 8:**
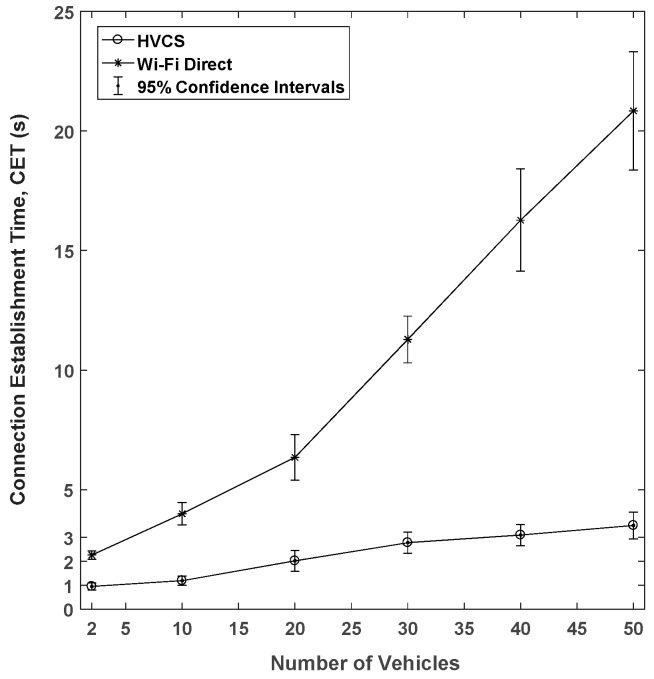
Comparison between the connection establishment time of the proposed HVCS and Wi-Fi Direct.

By using Veins, we measured the CET when there were more vehicles than that of the preliminary experiments for our prototype on the real road, as described in Section 4.1. CET indicates the total time of the connection and security establishment until data transmission is possible and is defined as follows.

(7) $$CET=\;\frac{\sum_{i=1}^{n}\sum_{j=1}^{m}tCDD_{i}^{j}+tCP_{i}^{j}}{n},$$

where n is the number of nodes; m is the number of times of the connections; tCDD is the amount of time taken for the node discovery and LAG formation; and tCP is the amount of the security establishment time between nodes in the LAG. In this experiment, it was assumed that only a single channel was used in the 400 m × 400 m of the road network with one signalized intersection of four main roads. The road has two lanes and the vehicle moves at an average of 60 km/h. Each CET is measured while we increase the number of vehicles existing in the transmission range at the same time. This means that, in one experiment simulated for 5 min, the traffic controller monitored two nodes at once to determine a LAG. This experiment was repeated until the number of vehicles associated with the CDD was 50. From Equation (8) formulated in Section 5.2, since $\nu_{s}$ is given as 58 vehicles in the map of 400 m × 400 m, it corresponds to 86% of the vehicle density when $\nu_{c}\;$ is set to 50 vehicles. Since the transmission range of a vehicle was 200 m in this small road network, all of the generated vehicles could communicate messages to each other within it. Figure 8 shows the connection establishment time of the HVCS and Wi-Fi Direct. We can see that the CET of Wi-Fi Direct takes about 2.26 s with 1.5 s for the DD and 0.76 for the provisioning when two nodes attempt to establish a connection between them. In contrast, the CET of the proposed system showed only 0.95 s, which is 2.3 times faster than that of Wi-Fi Direct.

With the help of the traffic controller, the HVCS can quickly form connections between vehicles, and the CET is not greatly affected by the number of vehicles that exist at the same time. In particular, it turns out that ten vehicles can be the preferred solution for the bounded time required for connection establishment since the CET value suddenly becomes higher when the *x*-axis value is ten [47]. Therefore, it is expected that HVCS has good performance when the LAG is formed with ten or fewer vehicles.

### 5.2. Experimental Environment

**Figure 9:**
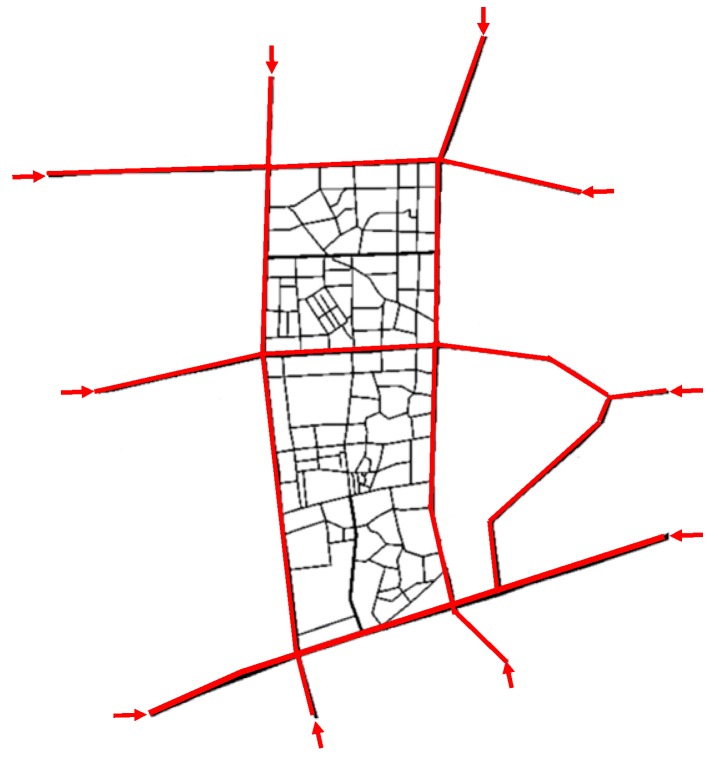
Road network.

In this subsection, we describe the environment for evaluating the performance of HVCS with a large traffic volume. We used some part of the road network of Daegu City in Korea. This experiment was performed using the road network with a size of 1000 m × 2000 m for two hours. The road network for this experiment corresponded to the map extracted from Open Street Map (OSM). Vehicles drove on the main road with the red lines and the alleyways with the black lines as shown in Figure 9, ten entry points of the main road are marked with red arrows. For each of the entrances, a vehicle was generated by a Poisson distribution with an average of 0.1 vehicles per second (λ=0.1) [48,49]. In two hours, a total of 2377 vehicles entered the road network. The maximum speed of the vehicle was given by the regulated speed of 60 km/h in Daegu. Vehicles were randomly assigned the entry road and the exit road, and traveled along a pre-selected path in the road network. 

Vehicle density and roadmap topology are important in vehicular network performance. In traffic engineering, the vehicle density is typically the number of vehicles present per unit space length, denoted as $\nu_{c}/l$, where $\nu_{c}$ is the number of vehicles occupying a length l of unit road section [50]. However, since the number of vehicles per unit length cannot accurately introduce the effect on the communication, the vehicle density should consider the total length of roads including the number and the length of the lanes present in a given area [51]. In this paper, to better identify the effect of the topology of the road network, the total length of roads considering the topology of our road network is represented using the number of vehicles in the situation where all of the roads are full of vehicles. The vehicle density δ is given as follows:

(8) $$\delta =\frac{\nu_{c}}{\nu_{s}}$$

where is the number of vehicles present in the road network and *V_s_* is the maximum number of vehicles that can exist in our road network. The number of vehicles at the time when the road network is full of vehicles is given by examining the number at the point where the ratio of incoming vehicles to outgoing vehicles to the road network becomes one. According to this, the vehicle density reached 100% when the number of vehicles became 600 in our road network.

A streaming event is defined as transmitting a video of a certain size from a source vehicle to a destination vehicle. In this simulation, since we designed it so that a streaming event was created uniformly every second starting 5 min after the first vehicle entered the road network, a total of 6900 streaming events were generated for two hours. A pair, consisting of the source and destination vehicles, was randomly selected for E2E video transmission. The data size of the event was designed based on the well-known and popular Netflix video streaming service as a benchmark. It defined a file size of 51.8 MB when transmitting one minute of video data with 25 FPS at 720p [52]. In order to turn video data into useful information for drivers, we set a file size of one streaming event to 100 MB corresponding to quality with 40 FPS at 720p. In the experiment, we employed a routing protocol of ad-hoc on-demand distance vector (AODV) using the cellular network to determine the end-to-end paths to transmit data. The SAF model was applied whenever the node did not connect immediately to the next-hop node. In Table 2, we summarize the experimental environment as discussed above.

### 5.3. Experimental Results

Using Veins, we evaluated the performance of the HVCS in terms of the throughput, transmission delay, propagation delay, E2E delay, and QoS. All the results of the HVCS were compared with those of WAVE. For reliable evaluation, the performance results were collected starting from 5 min after the first vehicle entered the road network until the end of the simulation time (2 h).

**Figure 10:**
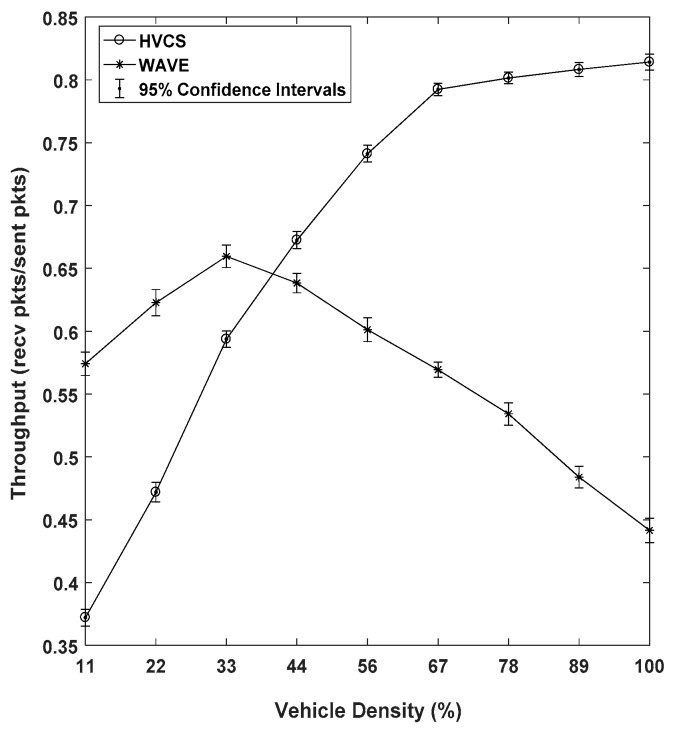
Throughput of the proposed system and the WAVE (the vehicle density was rounded off to the nearest whole number).

The throughput of the WAVE and our HVCS as a function of vehicle density is shown in Figure 10. At the vehicle density (≤33%) where the traffic amount is small relative to our road network size, the proposed system had lower throughput than WAVE. This is affected by the short transmission range (i.e., 200 m) of the HVCS. Therefore, the lower the vehicle density, the more isolated the vehicles. The isolation occurring at lower vehicle density may lead to performance degradation even though they perform local data propagation and SAF models. In the case of WAVE, it showed a considerable performance in a low-density region since it has a transmission range of 1000 m. As the vehicle density increased, the performance of HVCS overtook that of WAVE and reached about 80% at the vehicle density of 67%. The performance of WAVE started to drop after a vehicle density of 33%. This is because the wider communication range may cause more interference between vehicles on the network. 

**Figure 11:**
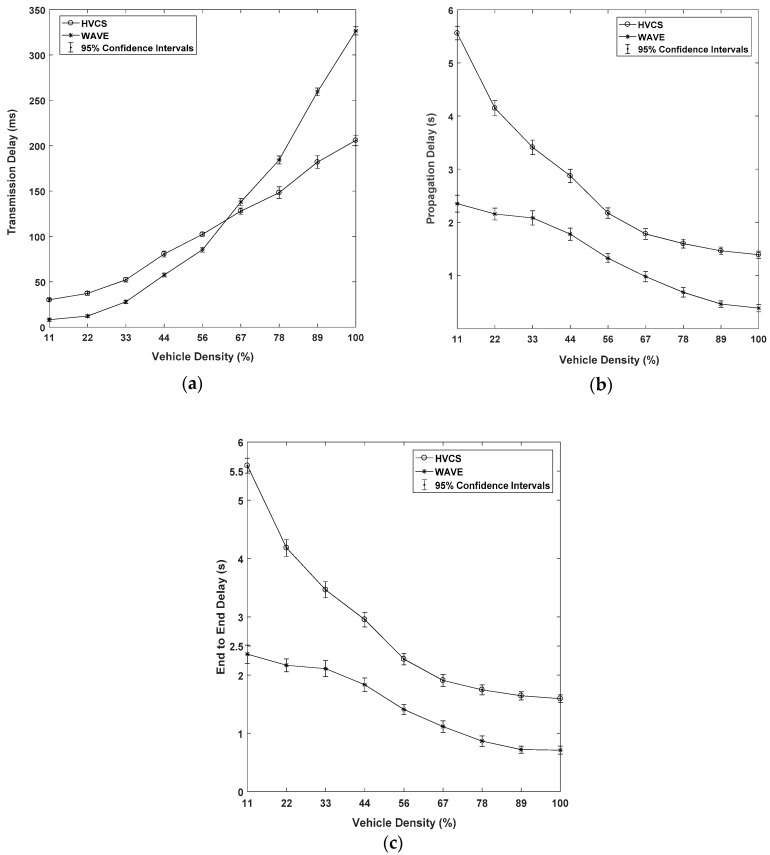
System connection performance (the vehicle density was rounded off to the nearest whole number): (**a**) Transmission delay; (**b**) Propagation delay; (**c**) End-to-end delay.

We show the results of the transmission, propagation, and E2E delays as a function of vehicle density in Figure 11. The E2E delay indicates the total time taken for a packet to arrive from the source node to the destination node. In other words, the E2E delay is defined as the sum of the transmission delay and the propagation delay. The transmission delay represents a level of connectivity within the LAG. The transmission delay is the time that each node takes to create a packet, access the channel, and send it to the next-hop node. As the vehicle density increases, the transmission delay linearly increases in the case of HVCS as shown in Figure 11a. However, the transmission delay of WAVE increased rapidly when the vehicle density was over 67%. This is because WAVE has a wider transmission range than the Wi-Fi Direct used for data transmission in the HVCS. For this reason, many vehicles experience interference. As shown in the graph, once the vehicle density exceeded 67%, the transmission delay of HVCS was lower than that of WAVE. 

The node carries out the SAF model when there is no next-hop node to send the data directly and immediately to neighboring vehicles. We defined the time taken for the SAF as the propagation delay. The results of the propagation delay of Figure 11b show the trend that the connectivity of the HVCS decreased and the delay increased at lower density. However, as the vehicle density became higher, it showed better performance. This indicates that the greater the vehicle density, the better the connectivity and the propagation delay is drastically reduced. In the case of WAVE, since its transmission range was longer than that of the HVCS, it achieved a short propagation delay. However, as the number of vehicles increases, collisions and interferences may increase. Therefore, we can see that the propagation delay does not decrease significantly.

In dynamic VANETs, it seems to be fact that the propagation delay is relatively large compared to the transmission delay. Therefore, the performance of the end-to-end multi-hop transmission is dependent on the propagation delay of the E2E delay. In this regard, it is not surprising that in Figure 11c, the trend line of E2E delay was similar to the line of the propagation delay. However, the result shows that the connectivity of the HVCS dramatically improves the E2E delay. 

In the case of a vehicle with one transceiver, it transmits and receives the non-safety data over WAVE while switching between the control channel and the service channel every 50 ms, according to the alternative channel access method in multi-channel operation. Even if there are four service channels in WAVE, the performance of WAVE is not better than that of Wi-Fi Direct because the time for transmission of the non-safety data is reduced by half and the data rate of Wi-Fi Direct is twice that of WAVE. Furthermore, even if some of the vehicles are distributed over multiple channels, the performance of transmitting a large volume of data in the WAVE with the wider range of transmission is highly affected as the vehicle density increases. In HVCS, it turns out that higher densities do not lead to degrading the efficiency of data delivery services as shown in Figure 10. The higher the density, the better the inter-vehicle connectivity (as shown in Figure 11b) and the less transmission redundancy and collisions caused by the channel contention (as shown in Figure 11a); hence, to support non-safety application over HVCS as an alternative to WAVE, vehicle density should be carefully considered to achieve acceptable performance.

**Figure 12:**
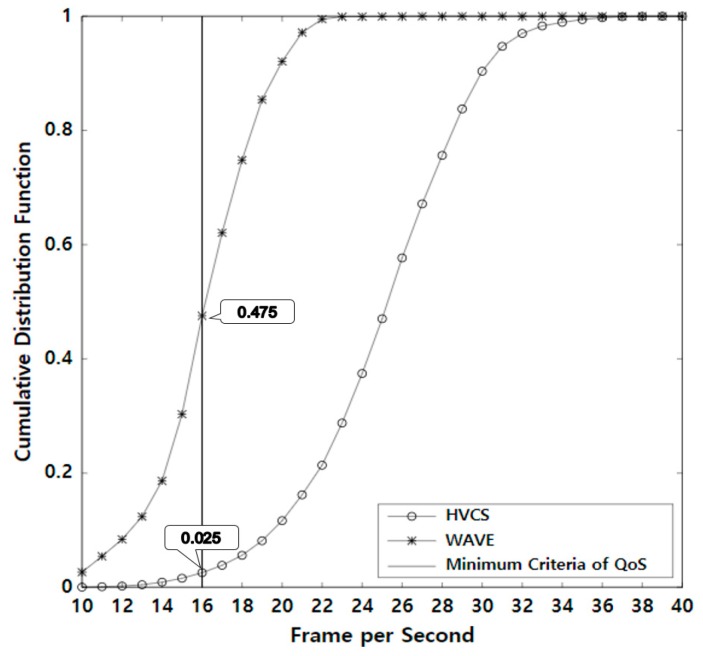
Quality of service level for the video streaming service.

We consider that our HVCS provides the timeliness of the real-time service if the end-to-end delivered video data satisfy the QoS requirement even if there is a slight delay. Hence, we checked if the proposed system satisfies the QoS of the video streaming service. It is known that a minimum of 16 FPS is required for recognizing consecutive images as a video [17]. In Figure 12, the average FPS of each streaming event during the experiment is shown as a cumulative distribution function. In the case of the proposed HVCS, the required QoS requirement was satisfied with 97.5% of the events played over 16 FPS. On the other hand, when WAVE was used, we saw that almost 50% of the streaming events did not satisfy the criterion of 16 FPS. We experimented to determine whether HVCS had the feasibility of delivering large volumes of data in qualitative aspects of the video service for VANETs. It was observed that the transmitted frames were available to play by examining the frames per second. Therefore, HVCS is capable of supporting a video streaming service over Wi-Fi Direct even in mobile environments while meeting the timeliness requirements.

## 6. Conclusions

In VANETs, there are various types of safety-critical and non-safety applications. People are concerned with convenience and pleasure, once they are satisfied with life-critical matters. For this reason, non-safety applications are important. In order to meet this demand, various types of communication technologies and devices will be used to organize the network. The non-safety applications will be realized in various forms according to the needs of consumers after the vehicular network is deployed in the near future. For the vehicular networks, we have studied the feasibility of V2X communication using another wireless technology, rather than using WAVE. 

As the core networking technology of C-ITS, WAVE is suitable for supporting safety-critical applications. However, it is difficult to guarantee its performance when transmitting non-safety data, especially high volumes of data, in a multi-hop manner. Therefore, we proposed a novel hybrid V2V communication system using a hierarchical networking architecture. It involves two models: the centralized control for fast connection establishment and the local data propagation to achieve reliability and efficiency. In the hierarchical networking architecture, the traffic controller is responsible for the centralized connection control that performs node discovery, group formation, and group management. The vehicle node is responsible for local data propagation within the group. To support the end-to-end multi-hop transmission over V2V communication, the store and forward model is employed. Under the centralized control, the CDD operates to improve the connection establishment time and the CP is performed to simplify the operations required for security establishment. We also implemented the proposed system exploiting both the cellular network and Wi-Fi Direct on both the smartphone and Windows system. The effect of the centralized control was shown in comparative experiments with Wi-Fi Direct. In the proposed system, the measured CET was only 0.95 s, that is, 2.3 times faster than that of Wi-Fi Direct. Using Veins, we demonstrated that the proposed system has high throughput and satisfies the QoS requirement of video streaming service even though the end-to-end delay is a bit longer, relatively than that of WAVE. The quality of the streaming service using WAVE was not acceptable. The proposed HVCS provides reliable and efficient transmission of large amounts of data through V2V communication to a given destination.

This paper demonstrates that Wi-Fi Direct designed for stationary environments can be used in mobile environments with the help of centralized control over cellular networks. The vehicle for local propagation can optimize the time spent to form a connection when the group is formed with up to ten vehicles. Our HVCS is useful for data transmission when the vehicle density is over 33%. For the non-safety applications in urban VANETs, our HVCS can coexist with WAVE and can become a surrogate of the WAVE. Moreover, the HVCS allows WAVE to be used as a dedicated networking technology to support only safety-critical applications.

In this paper, we focused on the non-safety application of video streaming, which generates a large amount of traffic as the communication range of Wi-Fi Direct increases in HVCS. However, it is important to create a framework that can operate any kind of application, not just one application, and work with all kinds of devices. Due to the scale of implementation, the functions to interact with other devices and applications have not been included in this work. We plan to extend our work as follows: 

We plan to develop the HVCS based on an actual device and try to make a system that can stream the CCTV data provided by the Korea Expressway Corporation or video data from the vehicles’ black box in real time. Using our system installed in the vehicle, we will perform a test on a road to obtain realistic results.

## Figures and Tables

**Table 1:** Comparison between Wi-Fi Direct and WAVE.

Parameters	Wi-Fi Direct	WAVE
Penetration rate	High	Low
Data rate	250 Mbps	27 Mbps
Tx range	Max 200 m	Max 1000 m
Connection establishment time	5~15 s	Immediate
Ad-hoc network	Limited	Support
Frequency	2.4 GHz~5 GHz	5.9 GHz

**Table 2:** Simulation parameters.

Parameter		Value
Map size		1000 m × 2000 m
Total number of vehicles generated		2377
Total number of streaming events		6900
Simulator		VEINS (ver. 3.0)
WAVE	Data rate	27 Mbps
	Transmission range	1000 m
	Transceiver	1
Wi-Fi Direct	Date rate	54 Mpbs
	Transmission range	200 m
	Transceiver	1
Total simulation time		2 h
The maximum speed		60 km/h
Routing protocol		AODV
Video data size		100 MB
